# Magnitude of opportunistic diseases and their predictors among adult people living with HIV enrolled in care: national level cross sectional study, Ethiopia

**DOI:** 10.1186/s12889-018-5733-x

**Published:** 2018-07-03

**Authors:** Teklu Weldegebreal, Ismael Ahmed, Abiyou Muhiye, Shoandagne Belete, Alemayehu Bekele, Mirgissa Kaba

**Affiliations:** 1 U.S. Centers for Disease Control and Prevention- Ethiopia, Addis Ababa, Ethiopia; 2 Ethiopian Public Health Association, Addis Ababa, Ethiopia; 3 School of Public Health, Addis Ababa University, Addis Ababa, Ethiopia

**Keywords:** Opportunistic diseases, HIV, Ethiopia, Magnitude, Predictors, Cross sectional study

## Abstract

**Background:**

Opportunistic diseases cause morbidity and mortality among human immunodeficiency virus (HIV) infected persons. There is dearth of evidence on the magnitude and predictors of opportunistic diseases among PLHIV in Ethiopia. This study was conducted to determine the magnitude and predictors of opportunistic diseases among adults enrolled in the national HIV/AIDS care and treatment services and generate information for program planning and medicine quantification in the country.

**Methods:**

A health facility-based cross-sectional study was conducted. Probability proportional to size and random sampling methods were employed to select health facilities and medical records of adult HIV-infected patients respectively. A total of 7826 medical records were reviewed from 60 health facilities nationwide. Socio-demographic and clinical data including diagnosis of opportunistic diseases were collected from the medical records. Period prevalence of opportunistic diseases over one year period was determined. Bivariate and multivariate logistic regression was used to measure associations between independent variables and the dependent variable, occurrence of opportunistic diseases.

**Results:**

Of the total of 7826 study participants, 3748 (47.9%) were from hospitals and 4078 were from health centers. The majority (61.8%) were female. The median age was 32 years with interquartile range (IQR) of 27–40. The median duration of stay in HIV care was 56 (IQR = 28–80) months; 7429 (94.9%) were on antiretroviral treatment. A total of 1665 cases of opportunistic diseases were recorded with an overall prevalence estimated at 21.3% (95% confidence interval (CI): 20.36, 22.18%). Skin diseases (4.1%), diarrhea (4.1%), bacterial pneumonia (3.6%), recurrent upper respiratory tract infections (3.1%) and tuberculosis (2.7%) were the leading opportunistic diseases. Isoniazid preventive therapy coverage among eligible patients was 24.8%. Persons with a CD4 count < 200 cells/mm^3^ [adjusted odds ratio (AOR) 1.80, 95% CI: 1.45, 2.23]; and who were bed ridden or ambulatory functional status [AOR (95% CI) = 3.19 (2.32, 4.39)] were independent predictors of diagnosis of opportunistic diseases.

**Conclusion:**

Opportunistic diseases were found to be pervasive among HIV infected adults in Ethiopia. Proactive identification and management, and prevention of opportunistic diseases should be strengthened especially among females, ambulatory or bed-ridden, and patients with low CD4 cell count.

## Background

Evidence on epidemiologic trend in low- and middle-income countries show a decline in the incidence and prevalence of opportunistic diseases following improvement in the quality of care services and the introduction of highly active antiretroviral treatment (ART) [1, 2]. Over the last decade, mortality among people living with human immunodeficiency virus (PLHIV) has decreased significantly [3]. In a study conducted at seven University-affiliated Hospitals in Ethiopia from January 2009–July 2013, mortality of 5.4/100 person-years of observation has been reported among adult PLHIV [4]. However, opportunistic diseases still remains the main cause of morbidity and mortality among PLHIV [5].

It is also evident that there is a disparity in the magnitude and types of opportunistic diseases between high- and low-income countries, and even between low-income countries themselves [6]. Thus, it is crucial for a country and a program to determine the types and magnitudes of opportunistic diseases affecting PLHIV in order to deliver impactful interventions and effectively manage resources.

Ethiopia has accomplished much in terms of HIV service expansion and uptake, which impacted on prevention and control of human immunodeficiency virus (HIV) and acquired immunodeficiency syndrome (AIDS) over the last decade. Ethiopian Public Health Institute (EPHI) estimated a total of 724,400 PLHIV with adult HIV prevalence of 1.2% for the year 2014 [7]. The Federal Ministry of Health (FMoH) 2014/2015 Annual Performance Report shows 375,811 PLHIV were enrolled and actively retained in the national ART program [8].

However, despite the progress, little evidence exists regarding the magnitude, and distribution of opportunistic diseases among PLHIV in Ethiopia. Available studies [9–12] and their estimates so far have been based on data from limited health facilities, small number of patients and varying time periods; thus limiting their generalizability and utility for national use. As a result, planning treatment and prevention interventions, developing and quantifying an evidence-based prioritized list of medicine and diagnostics has been a challenge. Therefore, this national level study was conducted to generate data on the type, magnitude and predictors of opportunistic diseases among adult PLHIV who were enrolled into the national HIV/AIDS care and treatment program. Information from this study will be used to facilitate program planning, and inform resource planning, specifically the quantification and distribution of medicines needed for management of opportunistic diseases in Ethiopia.

## Methods

### Study setting

Chronic HIV/AIDS care and treatment services in Ethiopia are provided at hospital and health center settings throughout the country. As of Dec. 2013, the FMoH adopted the 2013 World Health Organization (WHO) recommendation and moved ART eligibility criteria from ≤350 to ≤500 CD4 cell count irrespective of the WHO clinical stage. The chronic HIV/AIDS care service includes screening and management of opportunistic diseases. This study was conducted at national level by selecting representative public health facilities providing chronic HIV/AIDS care services irrespective of their geographic location using probability proportional to size (PPS) methodology.

### Study design

A health facility-based, cross-sectional study was carried out between September 2013 and August 2014. Medical records of HIV-infected adults who received pre-ART and/or ART care services in public health facilities in Ethiopia were reviewed.

### Study participants

The study participants were adult (age ≥ 15 years old) HIV-infected who were enrolled in care and received pre-ART or ART care and treatment services at least once between September 2013 and August 2014 in the selected public health facilities.

### Sample size and sampling procedure

The sample size was determined by employing the following formula and assumptions:

$$ n={Z_{\frac{a}{2}}}^2\left(\frac{P\left(1-P\right)}{d^2}\right) xD $$

P = Prevalence of clinically diagnosed Cryptococcus infection among PLHIV in Addis Ababa public hospitals was documented to be 3.7% [13].

d = margin of error between sample and population (0.006)

Z_α/2_ = critical value at 95% confidence interval (1.96)

D = design effect for a cluster sampling of hospitals and health centers (1.5)

Accordingly, sample size was estimated at 6267. During piloting of data collection procedures at two health facilities in Addis Ababa, we noted poor documentation of medical records. Therefore, an additional 30% allowance was made to compensate for poor documentation of medical records making the total sample size for the assessment to be 8147.

The number of health facilities and medical records needed to be reviewed from each sample facility was determined by considering the resources required to conduct the study and also calculating the limits of precision of the estimates. As a result, we determined that if 60–80 facilities are selected and higher than 75 medical records are reviewed from each facility, the precision of the estimate of these values will be ±0.006 or higher. Therefore, we decided to sample 60 health facilities and review 136 medical records from each of them.

**Fig. 1:**
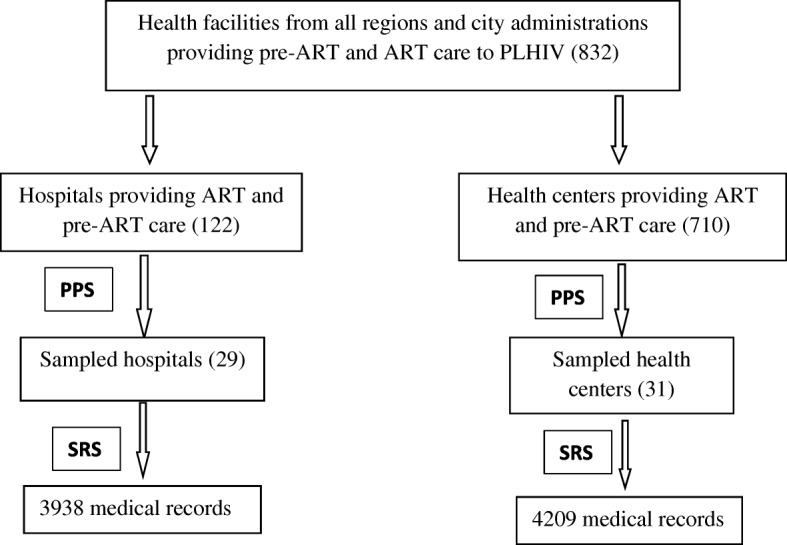
Schematic representation of sampling procedure

A two-stage, stratified sampling method was employed to sample the required health facilities and medical records. To select health facilities we used the President’s Emergency Plan for AIDS Relief (PEPFAR)-Ethiopia country office September 2013 report which listed 832 health facilities (710 health centers and 122 hospitals) with their PLHIV load from all regions of Ethiopia [14]. First, we prepared the sampling frame by stratifying health facilities into health center and hospital. Then, PPS sampling of facilities [15] was used to select health facilities from each stratum, independently; 31 health centers and 29 hospitals were selected using the PPS methodology. Medical records from each selected health facilities were selected using simple random sampling (SRS). Sample medical records were selected from electronic list of medical records (sampling frame) of pre-ART and ART patients in each of selected health facilities. Random numbers were generated using Excel sheet to select the required number of medical records from each facility. Schematics of sampling procedure is depicted in Fig. 1.

### Data collection tools and procedures

Prior to commencement of data collection, qualified data collectors and supervisors were recruited and trained on the contents of data abstraction tools, procedures and ethical standards; and data collection tools and procedures were field tested at two health facilities (1 hospital and 1 health center).

Upon arrival at the selected health facilities, the data collecting team met with ART program focal person and data management personnel and briefed them the study objectives and data collection processes. Data was collected from patients’ medical records by using a structured, paper-based, data abstraction tool. The tool captured data on socio-demographic variables including sex, age, education and marital status; clinical measurement including recent CD4 count, nutritional status, functional status and WHO clinical staging [16]; ART status and adherence to medications; diagnosis and treatment for opportunistic diseases; provision of isoniazid preventive therapy (IPT) and cotrimoxazole prophylaxis; and last documented follow-up status.

### Definitions of terms

*Nutritional status*: was determined by computing body mass index (BMI) as kg/m^2^. WHO classification was used to categorize participants: as severe thinness (BMI < 16.00 kg/m^2^), moderate thinness (BMI 16.00–16.99 kg/m^2^), mild thinness (BMI 17.00–18.49 kg/m^2^), normal (BMI 18.50–24.99 kg/m^2^), overweight (25.00–29.99 kg/m^2^) and obese (BMI ≥ 30.00 kg/m^2^). Patients with severe, moderate or mild thinness were categorized as undermatron.

*Functional status:* was defined in line with the definition on the national HIV care follow-up form: working functional status - able to perform usual work in and out of the house; ambulatory - able to perform activities of daily living but not able to work; and bedridden - not able to perform daily routine activities.

*Adherence*: was defined in line with the definition on the national HIV care follow-up form, adherence to ART was classified as good, fair or poor. Good adherence is defined as taking > 95% of prescribed doses, fair adherence as taking 85–94% of prescribed doses, and poor adherence as taking < 85% of prescribed doses. In this study, we defined good adherence to ART during the study period if the participant had constant good adherence status; fair adherence if the participant had at least one documented fair adherence status; and poor adherence if the participant had at least one documented poor adherence status throughout his/her follow-up during study period. Pre-ART and ART patients have follow-up visit to HIV clinic every six and three months on average, respectively.

*Follow-up status:* in line with the national HIV care follow-up form, a patient was considered lost to follow-up if the patient do not have a follow-up visit within 30 days after the latest clinic appointment date. If a patient moved to another health facility with confirmed written documentation of transfer out, follow-up was defined as transferred out. Death was ascertained only if documented in one of the reviewed medical records.

*Tuberculosis (TB) symptom screening* at last follow-up visit was used to categorize study participants into symptom screen result positive or negative. Occurrence of symptoms of cough, fever, night sweat and weight loss were the symptoms used for screening. Participants were categorized as symptom screen positive if they reported at least one of the above four symptoms during screening.

*Opportunistic diseases* were defined in accordance with the lists of opportunistic diseases indicated on the Ethiopian National Guideline for Comprehensive HIV Prevention, Care and Treatment [17]. The study only collected information on opportunistic diseases that were diagnosed between September 2013 and August 2014.

### Data management and quality assurance

Supervisors ensured adherence to standards of data collection at facility level by supervising data abstraction and checking for data quality in accordance with a standard data collection manual. Data quality issues identified onsite were addressed immediately. Data was checked for completeness and consistency, and double entered by two data entry clerks to ensure data quality.

### Data analysis

Data were entered into Epi Info software 3.5.1 to clean and ensure consistency of the data set. Clean data were exported to and analyzed using SPSS version 21. Descriptive statistics were used to analyze study participants socio-demographic and clinical parameters; the mean and standard deviation were calculated for normally distributed continuous variables and frequency (%) was used for categorical variables. Median and interquartile range was calculated for continuous variables with skewed distribution.

Opportunistic diseases documented on medical records of patients were used to analyze the types and frequencies of opportunistic diseases. The period prevalence of opportunistic diseases was determined as the proportion of adult PLHIV who developed one or more opportunistic diseases during the study period.

Bivariate statistics were employed to determine distribution of the study subjects by independent variable and to measure crude association between independent variables (sex, age group, recent CD4 count, taking prophylactic medications for opportunistic diseases, nutritional status, care status, follow-up status, and functional status) and the occurrence of opportunistic diseases. Odds ratios (OR) with 95% CI were used as a measure of association, and *p*-value of < 0.05 was considered statistically significant. Multivariate analysis was used to determine the independent effects of selected variables on opportunistic disease diagnosis controlling for the effects of others using complete case analysis. However, nutritional status measurement which was significantly affected by missing data (32%) was not included in the analysis. Independent predictor variables with a p-value < 0.05 were used for the final model and their association was calculated as adjusted odds ratio (AOR) with their 95% CI. Site-level clustering was not accounted in the analysis.

## Results

The socio-demographic characteristics of the study participants are presented in Table 1. The study reviewed medical records of 7826 adult PLHIV who had at least one follow-up visit at public health facilities in Ethiopia over the 12 months period, September 2013 to August 2014. The medical record retrieval rate was 96.1% of the planned records for review. Of the total participants, 3748 (47.9%) attended care and treatment services at hospitals and 4078 (52.1%) at health centers. The median age of study participants was 32 (IQR = 27–40) years. Females were relatively younger than males, median age of 30 and 36 years respectively. Nearly two third of participants (61.8%) were female. Among the 7109 with recorded marital status, more than half of participants 3612 (50.8%) were married and 2432 (34.2%) were either separated/divorced or widowed. Among the 7070 with recorded educational status, more than a third (34.9%) had no education, while 31.4% had secondary or more education.

**Table 1:** Socio-demographic characteristics of adult PLHIV in Ethiopia, September 2013 to August 2014

Characteristics	Number (%)
Health facilities where patients enrolled into care	
Hospital	3748 (47.9)
Health Center	4078 (52.1)
Total	7826 (100.0)
Sex	
Male	2989 (38.2)
Female	4837 (61.8)
Total	7826 (100.0)
Age group	
15–24	957 (12.2)
25–34	3365 (43.0)
35–44	2339 (29.9)
45–54	868 (11.1)
> 55	297 (3.8)
Total	7826 (100.0)
Marital status	
Never married	1065 (15.0)
Married	3612 (50.8)
Divorced/separated	1604 (22.6)
Wido*w*/widower	828 (11.6)
Total	7109 (100.0)
Education	
No education	2469 (34.9)
Primary	2384 (33.7)
Secondary	1662 (23.5)
Tertiary	555 (7.9)
Total	7070 (100.0)

### Clinical characteristics of study participants

The clinical characteristics of the study participants are presented in Table 2. The median time in HIV care was 56 (IQR = 28–80) months. Of the 7429 (94.9%) study participants on ART, 7346 (98.9%) were on first-line ART regimen and 83 (1.1%) were on second-line. The majority, 5689 (88.2%), of those participants who were on ART had consistent good adherence to their treatment throughout the follow-up period. Among the 7735 with recorded WHO’s clinical staging, 855 (11.1%) of clients were found to be at advanced clinical stages (stage III or IV). Of the 7730 with recorded functional status, 251 (3.2%) were either bed-ridden or ambulatory. Among the 5337 with recorded nutritional status, 1192 (22.3%) participants were undernourished. The median CD4 count for the total study population was 407 (IQR = 261–572) cells/mm^3^. Of the 6187 (79.1%) participants whose CD4 count was done and documented, 951 (15.4%) had CD4 count less than 200 cells/mm^3^.

**Table 2:** Clinical characteristic of adult PLHIV in public health facilities of Ethiopia, September 2013 to August 2014

Characteristics	Number (Percentage)
Care status	
On pre-ART care	397 (5.1)
On ART	7429 (94.9)
Total	7826 (100.0)
WHO clinical staging	
Stage I/II	6880 (88.9)
Stage III/IV	855 (11.1)
Total	7735 (100.0)
Functional status	
Working	7479 (96.8)
Ambulatory/bed ridden	251 (3.2)
Total	7730 (100.0)
Nutritional status	
Severe/moderate thinness	421 (7.9)
Mild thinness	771 (14.4)
Normal	3430 (64.3)
Overweight/obese	715 (13.4)
Total	5337 (100.0)
CD4 count (cells/mm^3^)	
< 200	951 (15.4)
200–349	1553 (25.1)
350–499	1556 (25.1)
≥500	2127 (34.4)
Total	6187 (100.0)
TB symptoms screening result	
Positive	428 (5.6)
Negative	7184 (94.4)
Total	7612 (100.0)
Isoniazid prophylaxis	
Yes	1703 (24.8)
No	5170 (75.2)
Total	6873 (100.0)
Adherence to ART	
Good	5689 (88.2)
Poor/fair	764 (11.8)
Total	6453 (100.0)
Cotrimoxazole prophylaxis	
Yes	5135 (97.1)
No	154 (2.9)
Total	5289 (100.0)
Median duration of stay in HIV care (months)	56 (IQR = 28–80)
Median CD4 count (cells/ mm^3^)	407 (IQR = 261–572)

TB symptom screening coverage at last visit was documented for 7612 (97.3%) participants; 7184 (94.4%) of screened participants had no TB symptoms. Of the 7184 with no TB symptoms, 6873 (95.6%) were eligible for IPT. However, IPT was provided for only 1703 (24.8%) of those eligible.

### Follow-up status of study participants in HIV care

**Fig. 2:**
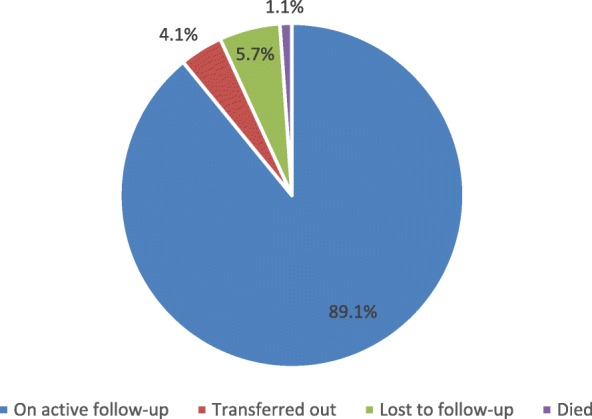
Follow-up status of adult PLHIV in health facilities of Ethiopia, September, 2013 to August, 2014

Last follow-up status was documented for 7794 (99.6%) of the participants. As shown in Fig. 2, 6944 (89%) of the participants were in active follow-up, 316 (4.1%) had been transferred out to other health facilities, 446 (5.7%) were lost to follow-up and 88 (1.1%) had died.

### Types and magnitudes of opportunistic diseases

A total of 1665 cases of opportunistic diseases were recorded for the study period, with a prevalence of 21.3% (95% CI: 20.36, 22.18%). We found 264 (3.4%) medical records which had documented chief complaints that may or may not associate with opportunistic diseases, but with no documented diagnosis. These medical records were treated as records with no opportunistic diseases diagnosis.

**Fig. 3:**
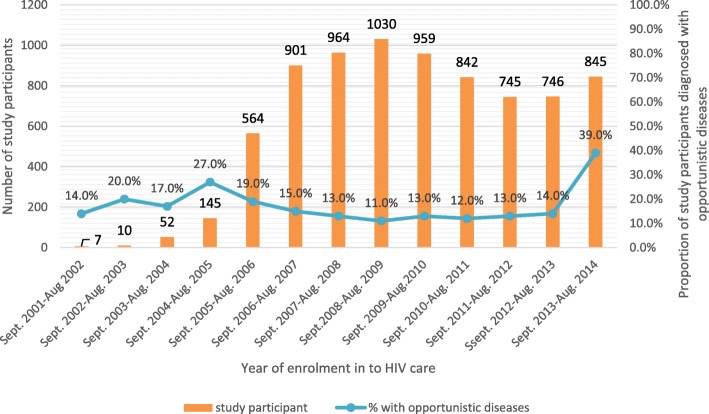
Proportion of adult PLHIV with OI by year of enrollment, September 2013 to August 2014

Of those diagnosed with opportunistic diseases 917 (55.1%) sought care at a hospital and 748 (44.9%) at a health centers. The proportion of participants with opportunistic diseases during study period categorized by year of their enrollment into the care service is shown in Fig. 3, the proportion of opportunistic disease among patients recently enrolled into HIV care was higher, estimated at 39%.

Types of opportunistic diseases diagnosed per patient ranged from one to six disease types: most (80.1%) had one type, 16.4% had two types and the remaining 2.3% had three and more types. The number of clinic visits per patient for diagnosis and management of opportunistic diseases ranged from one to eleven visits.

As shown in Table 3, skin diseases (4.1%), diarrhea (4.1%), bacterial pneumonia (3.6%), recurrent upper respiratory tract infections (3.1%) and TB (2.7%) were the leading opportunistic diseases. Severe forms of opportunistic infections were diagnosed more at hospitals than health centers: bacterial infections including bacterial meningitis and sepsis [OR (95% CI): 1.95 (1.23, 3.08)], bacterial pneumonia [OR (95% CI): 2.55 (1.98, 3.28)], and toxoplasma encephalitis [OR (95% CI): 9.25 (2.14, 40.00)]. Of a total of 206 TB cases, 160 (77.7%) were pulmonary TB, 26 (12.8%) were extra-pulmonary TB, and 20 (9.7%) were disseminated TB cases.

**Table 3:** Types and proportions of opportunistic diseases diagnosed among study participant during one year follow-up period, September 2013 to August 2014

Opportunistic Disease	Frequency (prevalence)	Frequency at hospital (prevalence)	Frequency at health Center (prevalence)	OR, at hospitals compared to health centers (95% CI)
Skin diseases	318 (4.1%)	169 (4.5%)	149 (3.7%)	1.13 (0.90, 1.43)
Diarrhea	317 (4.1%)	126 (3.4%)	191 (4.5%)	0.66 (0.52, 0.83)
Bacterial pneumonia	281 (3.6%)	197 (5.3%)	84 (2.1%)	2.35 (1.80, 3.05)
Recurrent upper respiratory tract infections	242 (3.1%)	113 (3.0%)	129 (3.2%)	0.88 (0.68, 1.14)
Tuberculosis	206 (2.7%)	98 (2.6%)	108 (2.6%)	0.91 (0.69, 1.20)
Oro-pharyngeal candidiasis	123 (1.6%)	82 (2.2%)	41 (1.0%)	2.00 (1.37, 2.92)
Severe bacterial infection	78 (1.0%)	50 (1.3%)	28 (0.7%)	1.79 (1.12, 2.85)
Perianal problems	31 (0.4%)	23 (0.6%)	8 (0.2%)	2.88 (1.28, 6.44)
Toxoplasma Gondi encephalitis	19 (0.2%)	17 (0.5%)	2 (0.1%)	8.50 (1.96, 36.84)
Pneumocystis pneumonia	13 (0.2%)	12 (0.3%)	1 (0.0%)	12.00 (1.56, 92.37)
Herpes simplex	12 (0.2%)	11 (0.3%)	1 (0.0%)	11.00 (1.42, 85.28)
Cryptoccoccal meningitis	8 (0.1%)	7 (0.2%)	1 (0.0%)	7.00 (0.86, 56.95)
Lymphoma	2 (0.0%)	1 (0.0%)	1 (0.0%)	1.00 (0.06, 16.00)
Other OIs	15 (0.2%)	11 (0.3%)	4 (0.1%)	2.75 (0.87, 8.65)
Total	1665 (21.3%)	917 (24.5%)	748 (18.3%)	1.23 (1.09, 1.37)

**Fig. 4:**
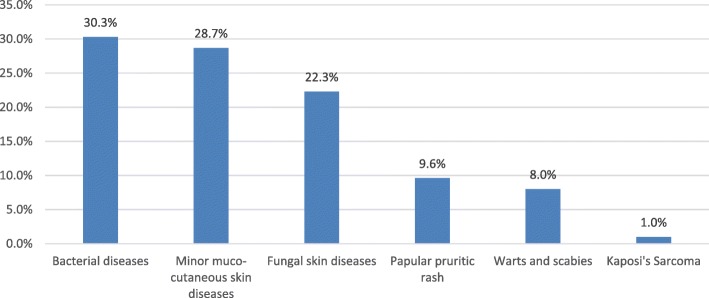
Distribution of skin diseases among adult PLHIV in Ethiopia, September, 2013 to August, 2014

As shown in Fig. 4, bacterial skin diseases (30.3%) such as cellulitis, impetigo and carbuncles were the most common skin diseases followed by minor mucocutaneous infections (28.7%).

### Predictors of opportunistic diseases

As shown in Table 4, in the multivariate logistic regression analysis participants with CD4 count < 200/mm^3^, sex, and ambulatory or bed-ridden functional status were independently associated with opportunistic diseases. Those with a CD4 count < 200 cells/mm^3^ were 80% more likely to have opportunistic disease compared to those with CD4 count ≥500 cells/mm^3^, [AOR (95% CI): 1.80 (1.45, 2.23)]; males were 14% less likely to have opportunistic diseases compared to females, [AOR (95% CI): 0.86 (0.74, 0.99)]; and PLHIV with either bed ridden and ambulatory functional status were 3.2 time more likely to be diagnosed with opportunistic diseases compared to those with working functional status, [AOR (95% CI): 3.19 (2.32, 4.39)].

**Table 4:** Predictors of opportunistic diseases among adult PLHIV, September 2013 to August 2014

Characteristics	COR (95% CI)	*P*-value	AOR (95% CI)	*P*-value
Sex (*n* = 7826)				
Male	0.917 (0.810, 1.038)	0.169	0.86 (0.74, 0.99)	0.047*
Female	1.00			
Age group (*n* = 7826)				
15–24	1.00			
25–34	0.81 (0.67, 0.98)	0.029*	0.83 (0.61, 1.03)	0.092
35–44	0.82 (0.68, 1.00)	0.052	0.89 (0.71, 1.12)	0.304
45–54	0.82 (0.64, 0.1.04)	0.104	0.80 (0.61, 1.07)	0.134
> 55	0.97 (0.70, 1.36)	0.879	0.93 (0.63, 1.38)	0.719
Recent CD4 (*n* = 6187)				
< 200	2.18 (1.81, 2.61)	0.001*	1.80 (1.45, 2.23)	0.001*
200-349	1.02 (0.85, 1.22)	0.851	0.99 (0.81, 1.21)	0.939
350–499	0.95 (0.79, 1.14)	0.556	0.94 (0.78, 1.14)	0.529
≥500	1.00			
Care status (*n* = 7826)				
In pre-ART care	1.72 (1.36, .2.18)	0.001*	1.33 (0.95, 1.85)	0.093
In ART	1.00			
Taking any prophylactic medication for opportunistic infection	1.00			
Yes	0.84 (0.74, 0.96)	0.008	0.92 (0.77, 1.08)	0.296
No				
Follow-up status (*n* = 7826)	1.00			
On active follow-up	1.11 (0.83, 1.50)	0.480	0.82 (0.56, 1.19)	0.292
Transferred-out	1.54 (1.22, 1.95)	0.001*	1.29 (0.93, 1.79)	0.125
Lost to follow-up	5.66 (3.71, 8.64)	0.001*	4.16 (2.26, 7.66)	0.001*
Died				
Recent functional status (*n* = 7730)				
Working	1.00			
Ambulatory or bed-ridden	4.75 (3.68, 6.13)	0.001	3.19 (2.32, 4.39)	0.001*

*Significant at *p*-value < 0.05

## Discussion

This cross-sectional study determined the common types of opportunistic diseases prevailing among PLHIV and their magnitude. Predictors for the occurrence of opportunistic diseases have also been outlined.

This study elucidated the proportion of opportunistic diseases affecting adult PLHIV in Ethiopia was 21.3% (95% CI- 20.36, 22.18%). This finding is comparable with 19.7% prevalence reported among adult PLHIV attending care services at Gondar University Hospital [9] and 22.4% prevalence reported from South East Nigeria [18]. However, it is lower than the prevalence reported from Hiwot Fana Hospital, 48.0% [10] and Debre Markos Hospital, 42.8% [11]. The observed difference between the current study and those reported higher prevalence could be due to differences in diagnostic capacity, study period or methods used to retrieve and analyze the data. The study from Hiwot Fana Hospital was not period specific while the study from Debre Markos Hospital reported on prevalence of opportunistic diseases during five to seven year on HAART follow-up and excluded diseases which patients initially presented with.

The current study also showed that proportion of opportunistic diseases among the newly enrolled adult PLHIV was higher than those who stayed in HIV care for a year or more. This could be because opportunistic diseases might have prompted patients to seek medical care at their initial visit or have manifested following enrollment in to care. The proportion (39.0%) observed in the current study was lower than the report from Felege Hiwot Hospital [12] which reported prevalence of 88.9% among ART-naïve adult PLHIV. The observed difference between the two studies could be due to changes in PLHIV care seeking behavior, access to ART services or the time period in which studies were conducted: in 2008 PLHIV were presenting to health facilities with advanced immune-compromise [mean (95% CI) for CD4 count: 153 cells/mm^3^ (139, 167)] and ART was offered for those with CD4 count less than 200 cells/mm^3^ as opposed to the current study. The current study also showed that the prevalence of opportunistic diseases among those stayed in care for more than seven years is relatively higher than those who were in care for more than a year but less than seven years; possibility of increased risk of ART failure in those group should be considered.

Regarding the types and magnitude of opportunistic diseases, the six major leading opportunistic diseases identified in this study were skin infections (4.1%), bacterial pneumonia (3.6%), recurrent upper respiratory tract infection (3.1%), tuberculosis (2.7%) and oro-pharyngeal candidiasis (1.6%). The pattern is different from the previous studies conducted in Northwest and Eastern part of Ethiopia [9–12]. This could be due to methodological differences with respect to operational definitions and also differences between study settings where all of the previous studies were single hospital-based studies. Our evaluation also showed differences in magnitude of those diseases between health facility types: bacterial pneumonia, skin diseases and diarrhea, consecutively, were the leading prevalent opportunistic disease among PLHIV attending at hospitals while diarrhea, skin diseases and recurrent upper tract infections were common among attendants at health centers. Moreover, it has elucidated that severe types of opportunistic diseases were less diagnosed in health center settings compared to hospitals. The differences between hospitals and health centers could be related with diagnosing capacity and quality of services, wherein hospitals have more doctors and greater diagnostic infrastructure compared to health centers, or it may be that sicker PLHIV tend to seek care more often at hospitals than health centers.

We found that skin diseases were the leading causes of morbidity in adult PLHIV in Ethiopia. Bacterial and fungal skin infections contributed 52.5% of the observed skin diseases. This finding was consistent with the study conducted in Brighton, department of HIV medicine [19] who reported infections as a leading causes of skin diseases among PLHIV followed by dermatosis, pruritus and malignancy; though the prevalence of skin diseases in their study was higher, 91.4%.

The proportion PLHIV diagnosed with of TB (2.7%) in this study is lower than reports from other similar studies which reported prevalence of 7.7% [18], 9.7% [9] and 18.2% [10]. Similar with a study conducted at Gondar University Hospital, [9] the majority of patients diagnosed with TB in this study had pulmonary form of the disease.

The current study found that despite high TB symptom screening coverage (97.3%), IPT service was provided only for 24.8% of eligible PLHIV; this finding is comparable to 19.6% IPT coverage reported from hospitals in Tigray Regional State of Ethiopia [20]. Drug availability and distribution, health care providers’ perception on importance of the service, and acceptability of the service among the PLHIV have been implicated as challenges to the program [21]. This low service coverage is a timely alarm to closely examine barriers to the IPT service and intensify the TB/HIV service package.

The study showed that low current CD4 count < 200 cells/mm^3^ was strongly associated with increased morbidity with opportunistic diseases, similar to studies from Ethiopia and other sub-Saharan Africa [9, 10, 18].

This study also showed that sex was independently associated with occurrence of opportunistic diseases. Female PLHIV were more likely to have opportunistic diseases compared to their male counterparts. Given female sex is disproportionately affected by HIV and majority (61.8%) of PLHIV in the care system are female [8] implications of this finding is paramount.

Lost to follow-up from the care services was found to be strongly associated with increases in the probability of diagnosis with opportunistic diseases. This finding is consistent with our observations in the health facilities, and studies reporting opportunistic diseases being among risk factors for lost to follow-up and deaths [4, 22–24].

Similar with a hospital-based study conducted at Hiwot Fana Hospital, Eastern part of Ethiopia [10], this study reports opportunistic diseases are significantly associated with PLHIV being bed ridden or ambulatory. This could be explained by lack or limited mobility of patients who may begin to lose interest in eating because they are not getting enough stimulation on a regular basis and unable to care for themselves.

PLHIV in pre-ART care had an increased association with opportunistic diseases as compared to those on ART, concordant with the study conducted at Gondar University Hospital [9]. In addition, the lost to follow-up rate was higher among PLHIV in pre-ART care (43.1%) compared to those on ART (3.4%); this finding is comparable to 38.4% lost to follow-up rate reported by a study that addressed the pre-ART care status in Ethiopia [22].

This national level cross sectional study was the first to our knowledge to establish national level information pertaining to opportunistic diseases among adult PLHIV enrolled in care. It has used a sufficient sample size with wider geographical coverage and health facility type representation to ensure generalizability; indicated the national level prevalence of opportunistic diseases, revealed the types, magnitude and predictors of opportunistic diseases as well as the coverage of critical care and support services; and estimated prevalence in a way that helps inform the procurement and distribution of medications for health facilities. However, due to the fact that this study was conducted using available medical records, retrospectively, it is subjected to limitations that emanate from incomplete recording of PLHIV clinical information on their records, and diagnostic capacity of health facilities involved in the study. Improvement in patients’ medical information documentations is one of the major issues the country health system need to work on. In addition to this, the OD identified in this study may not show the true picture of the common opportunistic infections prevalent among PLHIV in Ethiopia. This could be due to the fact that almost all of the study population were taking ART in addition to other OD preventing prophylaxis treatments.

This study has not assessed association of viral load status of participants with opportunistic diseases for the test was not performed routinely. Initiating and/or scaling up of routine viral load testing service at all ART service provision points is crucial to determine the association of viral load test result with opportunistic diseases and rate of treatment failure especially among PLHIV who have been on ART for long period of time.

## Conclusion

This study demonstrated that opportunistic diseases were pervasive among adult PLHIV enrolled in care in Ethiopia and distribution of opportunistic diseases was different from reports of previous studies conducted in the Eastern and Northwest part of Ethiopia. Future national level endeavors to prioritize quantification, procurement and distribution of medicines for the prevention and treatment of opportunistic diseases should take findings of this study into consideration.

CD4 count < 200/mm^3^, female sex, and ambulatory or bed-ridden functional status were independent predictors of opportunistic diseases. Opportunistic disease screening and prevention services should prioritize those PLHIV groups. Strategies and care models that emphasize early identification new PLHIV and accelerated implementation of test-and-treat approach including differentiated care models should be strengthened.

Only quarter of eligible PLHIV were on IPT. Importance of improving IPT service coverage in the country cannot be over emphasized. Strategies to mitigate challenges pertaining to IPT program and improve its service coverage should also be explored and implemented.

## Abbreviations

AIDS: Acquired Immunodeficiency Syndrome
ART: Antiretroviral Therapy
BMI: Body Mass Index
CDC: Centers for Disease Control and Prevention
EDHS: Ethiopia Demographic and Health Survey
EPHA: Ethiopian Public Health Association
EPHI: Ethiopian Public Health Institute
FMoH: Federal Ministry of Health
HIV: Human Immunodeficiency Virus
IPT: Isoniazid Preventive Therapy
OI: Opportunistic Infection
PEPFAR: President’s Emergency Plan for AIDS Relief
PLHIV: People Living with HIV
TB: Tuberculosis
WHO: World Health Organization
